# Shafting Misalignment Malfunction Quantitative Diagnosis Based on Speed Signal SVD-HT and CSF-PPSO-ESN Method

**DOI:** 10.1155/2022/7016597

**Published:** 2022-08-30

**Authors:** Zhen Yu, Wancheng Yu

**Affiliations:** ^1^Institute of Oceanographic Instrumentation, Qilu University of Technology (Shandong Academy of Sciences), 37 Miaoling Road, Qingdao 266001, China; ^2^Qingdao Industrial and Trade Vocational School, Qingdao 266041, China

## Abstract

Aiming at the quantitative diagnosis of shafting misalignment malfunction, a novel method based on speed signal with singular value decomposition and Hilbert transform (SVD-HT) and cubic spline fitting-Pareto particle swarm optimization-echo state network (CSF-PPSO-ESN) method is proposed. The malfunction diagnosis mechanism based on the speed signal is obtained by constructing the shaft misalignment malfunction model. Then, the SVD-HT and CSF-PPSO-ESN methods are applied to obtain the relationship between the shaft misalignment malfunction and the amplitude of the time and the rotation frequency (*f*_*r*_) component of the speed signal. The parameters of the CSF-PPSO-ESN method are settled according to the shaft misalignment malfunction and the *f*_*r*_ component of the speed signal. The accuracy of the proposed method is verified by using the *f*_*r*_ component of the speed signal and the trained CSF-PPSO-ESN to obtain the value of the shaft misalignment malfunction. The repeating experimental results show that the diagnosing error of the shaft misalignment malfunction can reach less than ±10 *μ*m. The method presented in this paper provides a novel way to diagnose shaft misalignment malfunction quantitatively.

## 1. Introduction

Misalignment malfunction will aggravate the vibration of the motor drive system, which will cause the problems such as drive shaft bending, base looseness, and bearing malfunction [1]. Moreover, the misalignment malfunction directly affects the transmission accuracy of precision instruments and even causes engineering accidents on some high-speed occasions [2]. Therefore, the misalignment malfunction diagnosis of the motor drive shaft system plays an essential role in ensuring the transmission accuracy of precision instruments, extending the service life of the equipment, and ensuring the safety of production operations [3–5].

It can be observed that an effective way of diagnosing, monitoring, and predicting the misaligned malfunction is necessary to improve the robustness and reliability of the designed measurement instrument. Multitudinous scholars have extensively studied the misalignment malfunction diagnosis based on vibration signals [6–8]. Wang et al. [9] proposed an information interval spectrum (IIS) malfunction diagnosis method to improve the diagnosis efficiency in a robust noise environment. Wang et al. [10] focused on sensitive feature extraction and pattern recognition of rolling bearing malfunction diagnosis and proposed an intelligent malfunction diagnosis method based on generalized composite multi-scale weighted permutation entropy, supervised Isomap (S-Iso), and marine predator algorithm machine. Tavasolipour et al. [11] discussed the problem of malfunction estimation for nonlinear systems with Lipschitz nonlinearities. Su et al. [12] proposed a hybrid method for rolling bearing malfunction diagnosis based on simulated annealing particle swarm optimization and an improved kernel-based extreme learning machine. Xu et al. [13] developed an improved multi-scale convolutional neural network (CNN) that integrates the feature attention mechanism model to solve the poor performance of traditional CNN-based models in unstable and complex working environments. Kim et al. [14] outlined a domain adaptive semantic clustering method to diagnose malfunctions in rotating machinery. In addition, because the noise in the signal affects the diagnosis and the separation of composite malfunctions, Meng et al. [15] proposed a method combining periodic weighted kurtosis—sparse denoising and periodic filtering to extract repeated pulses of composite malfunctions. Gonçalves et al. [16] proposed a pure output malfunction diagnosis method based on Markov parameters using random implementation theory. Chen et al. [17] proposed a malfunction diagnosis method for the long-term safe operation of rotating machinery based on improved composite multi-scale fuzzy entropy, topology learning, and out-of-sample embedding and support vector machine based on ocean predator algorithm. Zhang et al. [18] proposed a method based on hybrid attention improved residual network to diagnose malfunctions by highlighting the malfunction characteristics of the whole frequency band of wavelet coefficients and convolution channel.

Although the work of the scholars above based on vibration signals has achieved remarkable results, the drawbacks of those works are also apparent. First, additional errors may be introduced during the acquisition and processing of vibration signals, reducing the signal-to-noise ratio of the weak malfunction information in the original signal, leading to the failure of diagnosis [1, 2]. Secondly, due to the speed fluctuation of the shafting, the collected signal is usually nonstationary, or second-order cyclostationary and its characteristic parameters change with time [19, 20]. Moreover, the installation position of the sensor largely determines the quality of the collected signal [21, 22]. In addition, additional costs, environmental influences, and other factors also limit the development of vibration signal diagnosis methods [1]. So, it is necessary to find more suitable methods to diagnose malfunctions.

In recent years, scholars have gradually pursued electrical methods for their “nonintrusive” advantages. The electrical method realizes the system malfunction diagnosis by collecting signals such as phase current, electromagnetic torque, or speed and cooperating with signal processing technology [23]. Yang et al. [24] carried out malfunction diagnosis of motor bearing based on the characteristic analysis method of speed signal, which preliminarily showed the feasibility of the usual analysis method of the speed signal for mechanical malfunction diagnosis. Chandra and Sekhar [25] detect the coupling misalignment based on the torque signal. The effect of torque signals in misalignment malfunction diagnosis was verified. Prakht et al. [26] diagnosed and identified the misalignment malfunction based on the starting current signal of the motor.

However, both the method using current signal and the method using torque signals have disadvantages. The acquisition of the current signal will be disturbed by the sampling bias of the embedded system, current fundamental frequency component, and harmonics [1]. Besides, the order spectrum of the torque signal may contain periodic noise signals, making the malfunction signal not dominant in the spectrum, resulting in interference to malfunction diagnosis. In comparison, collecting the speed signal through the encoder has the advantages of convenient acquisition, higher sampling accuracy, and lower cost. At the same time, the diagnosis method based on the speed signal can realize real-time status monitoring [26].

Based on the above description, the misalignment malfunction diagnosis based on the speed signal has a broader application prospect. In addition, some optimization methods should be combined with speed signal analysis to diagnose the values of misaligned malfunction quantitatively. Multitudinous scholars have extensively studied optimization methods to analyze misaligned malfunction quantitatively. Cruz et al. [27] proposed a convolutional neural network based on an evolutionary algorithm. The technique consists of five convolutional neural networks, whose outputs are combined through a voting policy. Tirkolaee et al. [28] developed a Pareto-based algorithm combined with the NSGA-II and MOSA. NSGA-II is considered the most effective solution tool, and the solution's efficiency is increased by 20% by using this method. He et al. [29] combined the Pareto algorithm with the problem reformulation. The approach's signiﬁcant improvement beneﬁted from its performance and computational efﬁciency. However, all the above techniques focus on improving the Pareto optimal set, and their effects on the quantitative diagnosing of the misaligned malfunction are limited. Besides, these methods may have problems such as overfitting, falling into local optimization, and inability to converge.

The research on shafting misalignment malfunction diagnosis based on speed signal was carried out in this paper. Combined with the singular value decomposition and Hilbert transform (SVD-HT) method and cubic spline fitting-Pareto particle swarm optimization-echo state network (CSF-PPSO-ESN) method, a novel misalignment malfunction diagnosis method was proposed. The motor drive system is applied as a transducer to collect the speed signal. Compared with the typical vibration, current, and torque signal analysis, the method proposed in this paper eliminates the redundant transducers.

The proposed method can be used to quantitatively diagnose the values of misaligned malfunction for any rotation machine. Furthermore, the SVD-HT and CSF-PPSO-ESN methods are applied to simulate the relationship between the amplitude of the one time the rotation frequency (*f*_*r*_) component of the speed signal and the shaft misalignment malfunction. Then, the value of the shaft misalignment malfunction can be obtained using the amplitude of the *f*_*r*_ component and the CSF-PPSO-ESN. Based on the literature review, it can be seen that the novelty and progress beyond state of the art are that the method described in this paper not only has high prediction accuracy and accurate prediction value but also has a short running time for the algorithm.

## 2. Misalignment Malfunction Model and Diagnosis Mechanism

Misalignment mainly includes parallel misalignment, angular misalignment, and comprehensive misalignment. Considering that comprehensive misalignment combines parallel and angular misalignment, the following two types will be described in detail.

### 2.1. Parallel Misalignment Malfunction

**Figure 1:**
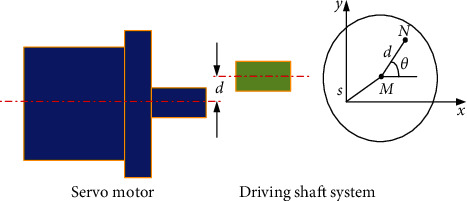
The parallel misalignment malfunction model.

Parallel misalignment malfunction refers to the phenomenon that the drive motor shaft and the shaft system are parallel but do not overlap [26]. The parallel misalignment malfunction model is shown in Figure 1.

In Figure 1, *M* is the center of the motor shaft, and N is the center of the drive shaft. The misalignment between the centers of the motor shaft and the drive shaft indicates a misalignment malfunction, *θ* is the angular position of the drive shaft, and *d* is the displacement of parallel misalignment.

Assuming that the speed of the transmission system is stable, the following formula is satisfied:

(1) $$\begin{array}{c} \theta =\omega_{m}t+\varphi , \end{array}$$

where *θ* is the angular position of the drive shaft; *ω*_*m*_ is the rotational angular velocity of the drive shaft; *t* is the time; and *φ* is the initial angular position.

The expression of the motor drive torque *T*_*m*_ can be obtained as shown in [27]

(2) $$\begin{array}{c} T_{m}=md\left[\ddot{x}\sin\;\theta -\left(g+\ddot{y}\right)\cos\;\theta \right]+T_{l}, \end{array}$$

where *m* is the equivalent mass of the rotor system; *g* is gravitational acceleration; *x* is the displacement of the motor axis in the *X*-direction; *y* is the displacement of the motor axis in the *Y*-direction; and *T*_*l*_ is the load torque of the rotor system.

It can be seen from the driving torque equation (2) that when there is a parallel misalignment malfunction in the system, the electromagnetic torque of the motor must not only meet the requirements of the load torque *T*_*l*_, but also overcome the fluctuating torque *T*_*C*_ introduced by the malfunction. The equation of *T*_*C*_ is as follows:

(3) $$\begin{array}{c} T_{C}=md\left[\ddot{x}\sin\;\theta -\left(g+\ddot{y}\right)\cos\;\theta \right]. \end{array}$$

To analyze the frequency components in fluctuating torque, combined with equation (1), equation (3) can be simplified to

(4) $$\begin{array}{c} T_{C}=A\;\cos\left(\omega_{a}t+\varphi \right). \end{array}$$

In equation (4), *ω*_*a*_=2*πf*_*r*_, where *f*_*r*_ is the rotation frequency; *A* is the constant coefficient. It can be seen that the fluctuating torque *T*_*C*_ is a sinusoidal signal of 1 time the frequency *f*_*r*_.

(5) $$\begin{array}{c} T_{C}=\mu_{1}k_{1}r_{1}d+\mu_{1}m_{1}r_{1}d\omega_{m}^{2}cos\left(\omega_{a}t+\varphi \right), \end{array}$$

where *μ*_1_ is the friction coefficient, *r*_1_ is the radius of the shaft, *k*_1_ is the elastic coefficient of the shaft, *d* is the misalignment value, and  *m*_1_ is the eccentric mass of the shaft.

Then, the relationship between the amplitude of the *f*_*r*_ component and the misalignment value can be obtained. That is,

(6) $$\begin{array}{c} d=\frac{A\;\cos\left(\omega_{a}t+\varphi \right)}{B\omega_{m}^{2}cos\left(\omega_{a}t+\varphi \right)+C}, \end{array}$$

where *B* and *C* are the constant coefficients.

Based on the above analysis, the diagnosis mechanism of parallel misalignment can be obtained: parallel misalignment malfunction will introduce a fluctuating torque of 1 time the rotation frequency into the load torque, and the electromagnetic torque will produce a fluctuating torque against it. The electromagnetic torque fluctuation will introduce the speed fluctuation of the same frequency into the motor speed signal. That is, the amplitude of the *f*_*r*_ component will increase in the frequency spectrum analysis of the speed signal. We can conclude that there is a nonlinearly relationship between the amplitude of the *f*_*r*_ component of the speed signal and the shaft misalignment malfunction. Then, the amplitude of the *f*_*r*_ component of the speed signal can be used to diagnose the parallel misalignment malfunction.

### 2.2. Angular Misalignment Malfunction

**Figure 2:**
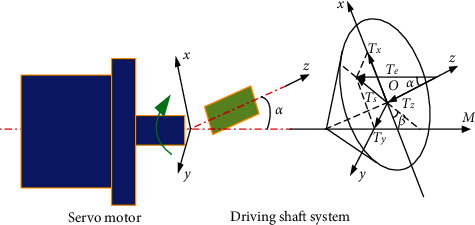
The angular misalignment malfunction model.

Angular misalignment malfunction refers to the premise that the drive shaft system axis and the axis of the motor shaft intersect at one point but are not parallel to each other, and there is a certain angle between the axis of the motor shaft and the axis of the drive shaft [26]. The angular misalignment malfunction model is shown in Figure 2.

As shown in Figure 2, the deflection angle formed between the motor shaft and the rotating shaft is *α*. The *Z*-axis coincides with the transmission shaft axis, and the motor torque *T*_*e*_ can be decomposed into the torque component *T*_*z*_ driving the rotating shaft along the *Z*-axis and the bending moment component *T*_*s*_ perpendicular to the rotor shaft. Moreover, it has the following relationship [30]:

(7) $$\begin{array}{c} T_{s}=T_{e}\sin\;\alpha =J_{r}{\ddot{\varphi }}_{r}, \end{array}$$

where *J*_*r*_ is the moment of inertia of the rotor system; *φ*_*r*_ is the rotation angle of the rotor, and *φ*_*r*_=*ω*_*r*_*t*+*θ*_*r*_; *ω*_*r*_ is the rotation angular velocity of the rotor, and $\omega_{r}={\dot{\varphi }}_{r}$; and *θ*_*r*_ is the rotation angular displacement of the rotor.

For the motor rotor system with the deflection angle *α*, the angular velocities of the two satisfy [30]

(8) $$\begin{array}{c} \frac{\omega_{r}}{\omega_{m}}=\frac{C}{1+D\;\cos\left(\varphi_{m}\right)}, \end{array}$$

where *ω*_*m*_ is the angular velocity of the motor; *φ*_*m*_ is the rotation angle of the motor, *φ*_*m*_=*ω*_*m*_*t*+*θ*_*m*_, and $\omega_{m}={\dot{\varphi }}_{m}$; *θ*_*m*_ is the torsion angle of the motor; *C* is the coefficient, *C*=4cos*α*/[3+cos(2*α*)]; and *D* is the coefficient, *D*=(1 − cos*α*)/[3+cos(2*α*)].

The Taylor expansion is

(9) $$\begin{array}{c} \frac{\omega_{r}}{\omega_{m}}=A_{0}+\sum_{n=1}^{\infty }{\left(-1\right)}^{n}A_{2n}\cos\left(n\omega_{m}t\right), \end{array}$$

where

(10) $$\begin{array}{c} \left\{\begin{array}{c} A_{0}=C\left(1+\frac{D^{2}}{2}+\frac{3D^{4}}{8}+\frac{5D^{6}}{16}+\frac{35D^{8}}{128}+\cdots \right)\text{,}\\ A_{2}=C\left(D+\frac{3D^{3}}{4}+\frac{5D^{5}}{8}+\frac{35D^{7}}{64}+\cdots \right)\text{,}\\ A_{4}=C\left(\frac{D^{2}}{2}+\frac{D^{4}}{2}+\frac{15D^{6}}{32}+\frac{7D^{8}}{16}+\cdots \right). \end{array}\right. \end{array}$$

Differential processing to equation (9),

(11) $$\begin{array}{c} \frac{{\dot{\omega }}_{r}}{\omega_{m}^{2}}=\sum_{n=1}^{\infty }{\left(-1\right)}^{n+1}B_{2n}\sin\left(n\omega_{m}t\right), \end{array}$$

where *B*_2_=2*A*_2_, *B*_4_=4*A*_4_, and *B*_6_=6*A*_6_.

Since ${\dot{\omega }}_{r}={\ddot{\varphi }}_{r}$, substituting equation (11) into equation (7), the fluctuating torque can be obtained as shown in

(12) $$\begin{array}{c} T_{s}=\frac{J_{r}\omega_{m}^{2}}{\sin\;\alpha }\sum_{n=1}^{\infty }{\left(-1\right)}^{n+1}B_{2n}\sin\left(n\omega_{m}t\right). \end{array}$$

In the existing system, since the torsional vibration amplitude of the high-frequency multiplier is weak and can be approximately ignored, the sinusoidal component of double-conversion frequency is mainly considered, so equation (12) can be further simplified.

(13) $$\begin{array}{c} T_{s}=\frac{J_{r}\omega_{m}^{2}}{\sin\;\alpha }\;B_{2}\sin\left(\omega_{m}t+\varphi_{m}\right). \end{array}$$

In equation (13), *ω*_*m*_=2*πf*_*r*_, where *f*_*r*_ is the rotation frequency. It can be seen that the fluctuating torque *T*_*s*_ is a sinusoidal signal of the *f*_*r*_ component, and there is a nonlinearly relationship between the two.

(14) $$\begin{array}{c} T_{s}=\mu_{1}k_{1}r_{1}sin\alpha +\mu_{1}m_{1}r_{1}tan\alpha \omega_{m}^{2}cos\left(\omega_{m}t+\varphi_{m}\right), \end{array}$$

where *μ*_1_ is the friction coefficient, *r*_1_ is the radius of the shaft, *k*_1_ is the elastic coefficient of the shaft, *α* is the angular misalignment value, and  *m*_1_ is the eccentric mass of the shaft.

Then, the relationship between the amplitude of the *f*_*r*_ component and the misalignment value can be obtained. That is,

(15) $$\begin{array}{c} sin\alpha =\frac{J_{r}\omega_{m}^{2}\cos\;\alpha B_{2}\sin\left(\omega_{m}t+\varphi_{m}\right)}{\sin\;\alpha C_{2}\omega_{m}^{2}\;cos\left(\omega_{m}t+\varphi_{m}\right)+A_{2}\sin\;\alpha \;\cos\;\alpha }, \end{array}$$

where *A*_2_ and *C*_2_ are the constant coefficients.

Based on the above analysis, the diagnosis mechanism of angular misalignment malfunction can be obtained: angular misalignment malfunction will introduce a fluctuating torque of 1 time the rotation frequency into the load torque, and the electromagnetic torque will produce a fluctuating torque against it. The electromagnetic torque fluctuation will introduce the speed fluctuation of the same frequency into the motor speed signal. That is, the amplitude of the *f*_*r*_ component will increase in the frequency spectrum analysis of the speed signal. We can conclude that there is a nonlinearly relationship between the amplitude of the *f*_*r*_ component of the speed signal and the shaft misalignment malfunction. Then, the amplitude of the *f*_*r*_ component of the speed signal can be used to diagnose the angular misalignment malfunction.

## 3. Signal Processing Method

The order spectrum analysis of the speed signal can be used to obtain the frequency spectrum. However, the order spectrum of the rotating signal may contain periodic noise signals, so the malfunction signal does not dominate in the frequency spectrum, thereby interfering with the malfunction diagnosis. The SVD-HT method is applied to the data processing to obtain the characteristics of the speed signal under different misalignment malfunction states.

### 3.1. Signal Processing Principle of SVD

For data processing, removing the direct current (DC) component in the speed signal is first necessary. The SVD filtering stage is included in the data processing of removing the DC component of the speed signal.

The SVD filtering algorithm uses the Hankel matrix structure to decompose the speed signal into a series of signal subspaces [31]. Assume that the speed signal *X*={*x*_1_,  *x*_2_,…,  *x*_*N*_}, where 1, 2,…, *N* are sampling points, respectively. The speed signal can be written in the matrix form shown in the following equation:

(16) $$\begin{array}{c} A=\left[\begin{array}{cccc} x_{1} & x_{2} & \cdots & x_{n}\\ x_{2} & \;x_{3} & \cdots & x_{n+1}\\ \vdots & \vdots & \vdots & \vdots \\ x_{m} & x_{m+1} & \cdots & x_{m+n+1} \end{array}\right]. \end{array}$$

In the above equation, the range of *n* is 1 < *n* *<* *N*, *m* = *N* − *n* + 1. By orthogonal transformation of matrix **A**, two standard orthogonal matrices **U** and **V** and diagonal matrix **D** can be obtained, where **U** = (**u**_1_, **u**_2_,…, **u**_*m*_) and **V**=(**v**_1_, **v**_2_,…, **v**_*n*_) ∈ *R*^*n*×*n*^*v*_1_**v**_2_**v**_*n*_ are orthogonal matrices and **D** = (*σ*_1_, *σ*_2_,…, *σ*_*r*_), and *σ*_1_ ≥ *σ*_2_ ≥ … ≥ *σ*_*r*_ ≥ 0; *r* = min (*m*, *n*) is the rank of matrix **A**. Then, the singular value decomposition of matrix **A** can be expressed as

(17) $$\begin{array}{c} A=UDV^{T}=\left[u_{1}u_{2}\cdots u_{r}\right]\left[\begin{array}{cc} \sigma_{1} & 0\;\cdots \;0\\ 0 & \sigma_{2}\;\cdots \;0\\ \vdots & \vdots \;\vdots \;\vdots \\ 0 & 0\;\cdots \;\sigma_{\text{r}} \end{array}\right]\left[\begin{array}{c} v_{1}^{T}\\ v_{2}^{T}\\ \vdots \\ v_{r}^{T} \end{array}\right]. \end{array}$$

Equation (17) can also be expressed as

(18) $$\begin{array}{c} D=\sum_{i=1}^{r}\sigma_{i}u_{i}v_{i}^{T}=\overset{r}{\underset{i=1}{\sum }}\sigma_{i}D_{i}, \end{array}$$

where *r* is the rank, *σ*_*i* _ are the singular values or weights with *σ*_1_ ≥ *σ*_2_ ≥ *···* ≥ *σ*_r_ > 0, and each **D**_*i*_ (or *σ*_*i*_) corresponds to singular vectors **u**_*i*_ and **v**_*i*_.

Singular values *σ*_1_,  *σ*_2_,…, *σ*_*r*_ are responses to singular values of different frequency components. The singular value can be used to obtain a reasonable, effective rank of various frequency components. The maximum singular value corresponds to the DC component, and the smaller singular value corresponds to the fluctuation signal caused by coupling misalignment malfunction.

Therefore, after the effective rank of the singular matrix is determined, the singular value with the maximum effective rank is eliminated. Then through the inverse operation of SVD, the matrix estimation of the DC component is obtained. Finally, the speed signal component caused by the coupling misalignment malfunction can be obtained through the inverse reconstruction of the phase space. The reconstructed speed signal components are *x*_*i*_ (*k*)(*k*=1,2,…, *m*). or (*k*=1,2,…, *n*) corresponding to order *i* which are obtained from the column or row vectors of *σ*_*i*_**D**_*i*_.

### 3.2. Signal Processing Principle of HT

After obtaining the speed signal components caused by the coupling misalignment malfunction using SVD filtering, the next step of signal processing is to use the HT to describe the variation law of the speed signal components' amplitudes with time and frequency in the whole frequency range. The results of HT are called the Hilbert spectrum, which reflects the periodic malfunction impact energy in mechanical malfunction diagnosis [32]. The HT $\hat{x}\left(t\right)$ of the speed signal component *x*(*t*) can be defined as

(19) $$\begin{array}{c} \hat{x}\left(t\right)=\frac{1}{\pi }\overset{+\infty }{\underset{-\infty }{\int }}\frac{x\left(t\right)}{t-\tau }d\tau =x\left(t\right)*\left(\frac{1}{\pi t}\right). \end{array}$$

Then, the analytical signals of the speed signal component can be constructed as follows:

(20) $$\begin{array}{c} \text{z}\left(\text{t}\right)=x\left(t\right)+j\hat{x}\left(t\right)=\text{a }\left(\text{T}\right){\text{e}}^{\mathrm{j\varphi}\left(\text{t}\right)}. \end{array}$$

The amplitude function *a*(*t*) of the speed signal component *x*(*t*) can be obtained by

(21) $$\begin{array}{c} a\left(t\right)=\sqrt{x^{2}\left(t\right)+{\hat{x}}^{2}\left(t\right)}. \end{array}$$

The phase function *φ*(*t*) of the speed signal component *x*(*t*) can be obtained by

(22) $$\begin{array}{c} \varphi \left(\text{t}\right)=\text{arctan}\frac{\hat{x}\left(t\right)}{x\left(t\right)}. \end{array}$$

Then, the instantaneous frequency of the speed signal component *x*(*t*) can be calculated as follows:

(23) $$\begin{array}{c} \omega \left(t\right)=\frac{d\varphi \left(t\right)}{dt}. \end{array}$$

After the process above, the Hilbert transform of each component can be expressed as

(24) Hω,t=Re∑i=1nAitei∫ ωitdt,

where Re represents the genuine part; *n* is the number of eigenmode functions; *A*_*i*_(*t*) is the *i*th component of the speed signal caused by the coupling misalignment malfunction; and *ω*_*i*_(*t*) is the instantaneous frequency of the *i*th component.

**Figure 3:**
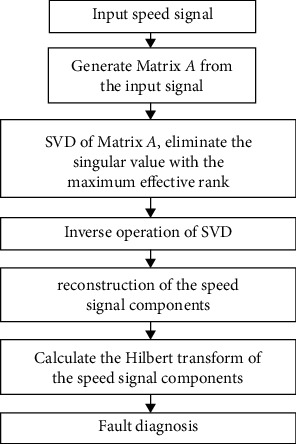
The scheme of signal processing.

After HT processing, the variation law of the amplitude of speed signal components with time and frequency in the whole frequency range can be accurately described. Combined with the frequency analysis of the speed signal component generated by the misalignment malfunction in Section 2, the type and value of misalignment malfunction parameters can be accurately diagnosed. It means that the type and value of misalignment malfunction parameters can be accurately diagnosed through the amplitude of the *f*_*r*_ component and other components in the SVD-HT spectrum of the speed signal. The scheme of signal processing is shown in Figure 3.

## 4. Proposed Malfunction Diagnosis Method

According to the analysis proposed in the second section, there is a nonlinearly relationship between the amplitude of the *f*_*r*_ component of the speed signal and the shaft misalignment malfunction. After obtaining the amplitude of the *f*_*r*_ component of the speed signal using the SVD-HT method, the relationship between the amplitude of the *f*_*r*_ component of the speed signal and the shaft misalignment malfunction can be obtained. Since the number of points that can be used to obtain the relationship is limited, it is necessary to use the limited points to get the relationship with high accuracy. Therefore, the novelty cubic spline fitting-Pareto particle swarm optimization-echo state network (CSF-PPSO-ESN) method is used to obtain the relationship with arbitrary accuracy and fully use discrete points. The CSF-PPSO-ESN method combines cubic spline fitting, Pareto particle swarm optimization, and echo state network. The method is described in detail in the following.

### 4.1. Cubic Spline Fitting Based on Pareto-PSO (CSF-PPSO)

#### 4.1.1. Cubic Spline (CS) Fitting

The coordinates of known points are set as (*x*_*i*_, *y*_*i*_)*i*=1,2,3,…, *n*. The so-called cubic spline fitting is to construct the cubic spline relationship between *y*_*i*_ and *x*_*i*_ according to the coordinates of known points. Through multiple iterations, the fitting residual is reduced as much as possible. Then, the optimal (minimum residual) cubic spline curve can be obtained, which can be used to estimate the coordinates of unknown points. The cubic spline equation is introduced in the following.

The general equation of the cubic spline curve is as follows:

(25) $$\begin{array}{c} Y\left(x\;\right)=\;\sum_{i\;=\;0}^{n}P_{i}y_{i,k}\left(x\right), \end{array}$$

where *P*_*i*_ is the coefficient of the cubic spline curve equation and *y*_*i*,*k*_(*x*) is the *k*-order cubic spline basis function.

The cubic spline basis function is as follows:

(26) $$\begin{array}{c} y_{i,k}\left(x\right)=\frac{1}{k!}\sum_{m=0}^{k-i}{\left(-1\right)}^{m}\left(\begin{array}{c} m\\ k+1 \end{array}\right){\left(x+k-m-j\right)}^{k}, \end{array}$$

where $\left(\begin{array}{c} m\\ k+1 \end{array}\right)$ denotes factorial, and the expansion of equation (26) is as follows.

(27) $$\begin{array}{c} y_{0,3}\left(x\right)=\frac{1}{6}{\left(1-x\right)}^{3},\\ y_{1,3}\left(x\right)=\frac{1}{6}\left(3x^{3}-6x^{2}+4\right),\\ y_{2,3}\left(x\right)=\frac{1}{6}\left(-3x^{3}+3x^{2}+3x+1\right),\\ y_{3,3}\left(x\right)=\frac{1}{6}\;x^{3}. \end{array}$$

The basis function in the cubic spline equation is substituted into equation (25) to obtain the following equation:

(28) $$\begin{array}{c} Y\left(x\;\right)=\;P_{0}y_{0,3}\left(x\right)+P_{1}y_{1,3}\left(x\right)+P_{2}y_{2,3}\left(x\right)+P_{3}y_{3,3}\left(x\right). \end{array}$$

Equation (27) is the cubic spline equat:on. The cubic spline equation should be established according to (6) and (15). The key to solving the cubic spline equation is to solve the value of the coefficient. The PPSO algorithm is used to optimize the parameters of the cubic spline equation used in this study.

#### 4.1.2. Particle Swarm Optimization Algorithm Based on Pareto Optimal Solution (PPSO)

This paper's model optimization algorithm [33] evolved from the particle swarm optimization (PSO) algorithm. In the PSO algorithm, each particle represents a solution of the cubic spline equation. Among all solutions, the best solution (with the smallest residual) is the best position, and each particle will find the best position in this region. In finding the best position, each particle will find the position closest to the best position, which is called the individual extremum. The global extremum is the best position of all particles in the search process. These particles will constantly adjust their speed and direction to approach the best position through these two positions. The updated formula of particle velocity and position is as follows:

(29) $$\begin{array}{c} v_{ij}^{m+l}=wv_{ij}^{m}+c_{1}r_{1}\left(p_{ij}^{m}-x_{ij}^{m}\right)+c_{2}r_{2}\left(g_{j}^{m}-x_{ij}^{m}\right)\text{,}\\ x_{ij}^{m+l}=x_{ij}^{m}+lv_{ij}^{m+l}. \end{array}$$

In the equations above, *i* = 1, 2,…, *n*, *j* = 1, 2,…, *j*, *m* = 1, 2,…, *M*. *m* is the number of iterations, *x*_*ij*_^*m*^ is the position of particle *i* in space, *v*_*ij*_^*m*^ is the velocity of particle *i* in space, and *p*_*ij*_^*m*^ and *g*_*j*_^*m*^ are defined as individual extremum and global extremum, respectively. *c*_1_ and *c*_2_ are acceleration coefficients, usually *c*_1_ = *c*_2_. *r*_1_ and *r*_2_ are random numbers in the interval [0, 1], and *w* is the weight expressed as follows:

(30) $$\begin{array}{c} w=\frac{w_{\max}-\left(w_{\max}-w_{\min}\right)\times m}{m_{\max}}. \end{array}$$

In the equation above, *m*_max_ is the maximum number of iterations, and *w*_max_ and *w*_min_ represent the maximum and minimum weights, respectively.

The Pareto optimal principle is introduced in this paper based on the PSO algorithm. The PPSO algorithm is used to obtain the solution set of the cubic spline model. The specific steps are as follows.

Firstly, the initial particle swarm in the nondominated solution set is estimated. The algorithm generates grids to divide the target space to be explored and establishes these grids into coordinate systems. In the coordinate system, the coordinates of each particle are defined according to the objective function value. In this way, the coordinates of each particle in the nondominated solution set can be located. The density value of each particle is the number of particles in the mesh where the particle is located. Then, the global and local search abilities are evaluated online by calculating the number of nondominated solutions in real time. When the particle's position *E*_*j*_, in the nondominated solution set, is selected as the best position of the particle *F*_*i*_ in the particle swarm, the fitness of the selection intensity is calculated by the following equations:

(31) $$\begin{array}{c} \text{Fit}\left(i,j\right)=\tau_{t}\cdot \frac{\max\left(\Delta \delta_{i}\right)}{\Delta \delta_{i,j}}+\left(1-\tau_{t}\right)\cdot \frac{\max\;\left(h_{j}\right)}{h_{j}},\\ \tau_{t}=\frac{m_{t}}{\left|E\right|},\\ \max\left(\Delta \delta_{i}\right)=\;\max_{j\in \left|E\right|}\left\{\Delta \delta_{i,j}\right\},\\ \max\;\left(h_{j}\right)=\;\max_{j\in \left|E\right|}\left\{h_{j}\right\},\\ \Delta \delta_{ij}=\left|\text{Sigma}\left(F_{i}\right)-\text{Sigma}\;\left(E_{j}\right)\right|+\varepsilon . \end{array}$$

In the equations above, *τ*_*t*_ represents the ability of global or local search, |*E*| represents the fixed size of the nondominated solution set *E*, *m*_*t*_ represents the number of members in the nondominated solution set in generation *t*, *h*_*i*_ is the number of members in the grid where *E*_*j*_ is located, Δ*δ*_*ij*_ represents the distance between the sigma value of *F*_*i*_ and the sigma value of *E*_*j*_, and *ε* is a small positive number. For the two-dimensional optimization problem, Sigma(*F*_*i*_) is calculated by the following formula [34]:

(32) $$\begin{array}{c} \text{Sigma}\left(F_{i}\right)=\frac{{\left(K_{2}f_{1}\right)}^{2}-{\left(K_{1}f_{2}\right)}^{2}}{{\left(K_{2}f_{1}\right)}^{2}+{\left(K_{1}f_{2}\right)}^{2}}, \end{array}$$

where *f*_1_ and *f*_2_ are the target value of *F*_*i*_, and *K*_1_ and *K*_2_ are the maximum value of *F*_*i*_'s first and second target values. Select the particle with the most significant fitness value as the best position for *F*_*i*_

As seen above, the ultimate goal of PPSO is to obtain the best position of the population of all particles. The cubic spline equation with a residual error of 10^−8^ can be obtained after iteration using the PPSO algorithm. Then, the coordinates of unknown points can be estimated according to the obtained equation.

### 4.2. Echo State Network (ESN)

#### 4.2.1. ESN Model

**Figure 4:**
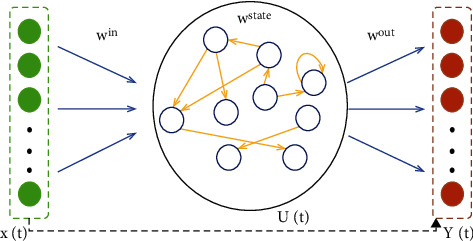
ESN structure diagram.

The ESN is a unique neural network that replaces the traditional hidden layer structure with a reserve pool with many randomly initialized neurons [35]. The ESN consists of the input layer, reserve pool, and output layer. The neurons of the three parts are *L*, *M*, and *N*, respectively. The neuron vector expressions of these three parts can be expressed by formulas (32)–(34).  Figure 4 shows the structure of the ESN.

(33) $$\begin{array}{c} X_{\left(p\right)}={\left(x_{1}\left(p\right),\;x_{2}\left(p\right),\;\cdots ,\;x_{L}\left(p\right)\right)}^{T}, \end{array}$$

(34) $$\begin{array}{c} U_{\left(p\right)}={\left(u_{1}\left(p\right),\;u_{2}\left(p\right),\;\cdots ,\;u_{M}\left(p\right)\right)}^{T}, \end{array}$$

(35) $$\begin{array}{c} Y_{\left(p\right)}={\left(y_{1}\left(p\right),\;y_{2}\left(p\right),\;\cdots ,\;y_{N}\left(p\right)\right)}^{T}. \end{array}$$

The input data *X*(*T*) are transferred to the reserve pool through *W*^in^'s connection matrix. The weight matrix in the reserve pool is *W*^state^. After training, the results are transmitted to the output layer through the output matrix *W*^out^. *W*^in^ and *W*^state^ are determined randomly during initialization, and their parameters are continuously optimized through training. It is worth noting that *W*^state^ should be a sparse matrix with a spectral radius of less than 1 to prevent the explosion of the reserve pool. In training, the internal state of the reserve pool is determined by equation (35).

(36) $$\begin{array}{c} U\left(p+1\right)=a\cdot f_{1}\left(W^{\text{in}}x\left(p+1\right)+W^{\text{state}}u\left(p\right)\right). \end{array}$$

Meanwhile, the model's output can be obtained from equation (36).

(37) $$\begin{array}{c} Y\left(p+1\right)=f_{2}\left(W^{\text{out}}\left(U\left(p+1\right),\;X\left(p+1\right)\right)\right), \end{array}$$

where a represents the ratio of *U*(*p*) and *U*(*p*+1), and its value range is [0, 1]. Functions *f*_1_ and *f*_2_ are activation functions, which are usually hyperbolic tangent functions. At this time, it is necessary to standardize the training data set before training to ensure that the model can calculate the output correctly. The activation function is shown in the following equation:

(38) $$\begin{array}{c} f_{1}\left(x\right)=f_{2}\left(x\right)=1-\frac{2e^{-x}}{e^{x}+e^{-x}}, \end{array}$$

where *W*^out^ is the matrix to be calculated, which can be calculated by formula (38).

(39) $$\begin{array}{c} W^{\text{out}}={\left(U^{T}U\right)}^{-1}U\cdot Y. \end{array}$$

#### 4.2.2. Key Parameters

The neurons of ESN are concentrated in the reserve pool, and the number of neurons determines the ability of the whole system model to deal with complex problems. Generally speaking, the more the neurons in the reserve pool, the stronger the model's ability to deal with high-dimensional spatial nonlinear problems and in computational simulation. However, if the number of neurons increases, the training time of the system will become longer. When the number of neurons reaches a certain threshold, the model will overtrain and waste time. Therefore, the number of neurons in the reserve pool should be set reasonably to avoid over-training.

Not every neuron in the reserve pool is connected to other neurons. SD is the proportion of neurons in the reserve pool that produce associations. SD can be obtained by equation (39).

(40) $$\begin{array}{c} \text{SD}=\frac{m}{M}, \end{array}$$

where *m* is the number of associated neurons in the reserve pool and *M* is the total number of neurons in the reserve pool. The greater the ratio of SD, the better the nonlinear approximation ability of the model.

Another parameter that needs attention is the spectral radius of the weights in the reserve pool. The spectral radius is the maximum eigenvalue of the weight matrix *W*^state^. The spectral radius can be calculated by equation (40).

(41) $$\begin{array}{c} \rho =\max\left\{\text{abs}\left[E\left(W^{\text{state}}\right)\right]\right\}, \end{array}$$

where *E*(*W*^state^) represents the eigenvalue of *W*^state^. The model remains stable when *ρ* < 1 and produces an “echo state.” If *ρ* is too large, the ESN will become unstable. The spectral radius of the system is randomly generated in the range of [−1, 1] to avoid instability.

*Is* is a scale factor. It is used to scale the input signal. The input signal is multiplied by the scale factor and then connected to the neurons in the reserve pool. The value of *Is* is related to the nonlinear strength of the relationship to be simulated. The stronger the nonlinearity, the greater the value. The above parameters are closely related to the performance of the ESN model. They will directly affect the model's performance if they are not set correctly.

### 4.3. CSF-PPSO-ESN Algorithm

As is described above, the number of sampling points is limited by the number of misalignment values. The fitted relationship model between the amplitude of the *f*_*r*_ component of the speed signal and the shaft misalignment malfunction cannot fully reflect the relationship characteristics between them when the traditional numerical fitting methods are used to fit the relationship between the two. Because of this, this paper combines the cubic spline fitting method based on PPSO with the ESN neural network method. Based on the CSF-PPSO-ESN method, the accuracy of misalignment malfunction diagnosis is further improved. The specific implementation steps of the CSF-PPSO-ESN combination fitting method are as follows.

**Step 1 .**

Measuring the amplitude data of the *f*_*r*_ component in multiple groups of speed signals with different initial misalignment values. The dynamometer is installed on the shaft system equipped with coupling. The misalignment between the initial motor and dynamometer shaft is 0 (±0.01 mm/°). Then, the misalignment of the initial motor shaft and dynamometer shaft is adjusted to 0.25 mm/°, 0.5 mm/°, 0.75 mm/°, 1 mm/°, 1.25 mm/°, 1.5 mm/°, 1.75 mm/°, and 2 mm/° (±0.01 mm/°). The experiment is carried out again to obtain the amplitude (*v*_*frk*_, *m*_*k*_ ). of the *f*_*r*_ component in the eight groups of speed signals. *k* = 1, 2,…, 8. The experiment is repeated five times to obtain the amplitude of the *f*_*r*_ component in the first group of speed signals.

**Step 2 .**

Fitting the relationship curve. The amplitude model *f*(*v*) of the shaft misalignment malfunction value is fitted based on the amplitude of the *f*_*r*_ component of the speed signal, and the shaft misalignment value obtained from the first group of the experiment using the cubic spline fitting method based on PPSO.

**Step 3 .**

Multi-group data prepossessing. The amplitude of the *f*_*r*_ component of the speed signal and misalignment value are used as the benchmark. Select the *f*_*r*_ component interval (*v*_*frk* +1_^1^, *m*_*k*_^1^) within which the amplitude model *f*(*v*) of the shaft misalignment value has a stable variation trend. Selecting the *f*_*r*_ component quantity (*v*_*frk*_^2^, *m*_*k*_^2^), (*v*_*frk*_^3^, *m*_*k*_^3^) falling in this interval in the other two groups of data, the adjustment coefficients *a*_*k*_^2^ and *a*_*k*_^3^ of group 2 and group 3 can be obtained using the fitting value *f*(*v*_*frk*_^2^), *f*(*v*_*frk*_^3^) of the misalignment value, and subtract the value *m*_*k*_^2^ and *m*_*k*_^3^  of the actual shaft misalignment, that is,

(42) $$\begin{array}{c} a_{k}^{2}=f\left(v_{frk}^{2}\right)-m_{k}^{2}\text{,}\\ a_{k}^{3}=f\left(v_{frk}^{3}\right)-m_{k}^{3}\text{.} \end{array}$$

Then, *a*_*k*_^2^ and *a*_*k*_^3^ are added to the value *m*_*k*_^2^ and *m*_*k*_^3^ in group 2 and 3 data, respectively, to obtain the shaft misalignment value. Then, the measurement data of each group could be correlated. That is,

(43) $$\begin{array}{c} m_{k}^{1^{\prime }}=m_{k}^{1}, \end{array}$$

(44) $$\begin{array}{c} m_{k}^{2^{\prime }}=m_{k}^{2}+a_{k}^{2}, \end{array}$$

(45) $$\begin{array}{c} m_{k}^{3^{\prime }}=m_{k}^{3}+a_{k}^{3}. \end{array}$$

**Step 4 .**

The prepossessing amplitude data of the *f*_*r*_ component speed signal misalignment (*v*_*frk*_,  *m*_*k*_′) are substituted with the neural echo state network. When used, the number of hidden layer neurons, learning rate, training method, and other echo state network neural network parameters are selected. The final relationship model between the amplitude of the *f*_*r*_ component in the speed signal and the misalignment is obtained. The value range of the number of reserve pool neurons is obtained according to equation (45), and the effect is estimated according to the fitted relationship model.

## 5. Experimental Tests

### 5.1. Test Rig

**Figure 5:**
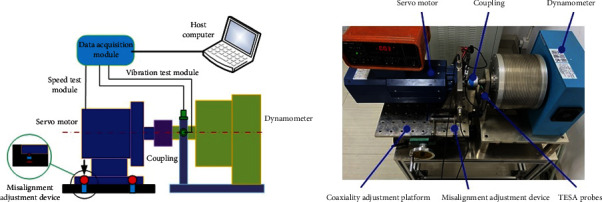
The test rig: (a) overall structure diagram; (b) physical drawings.

The overall structure diagram and physical drawings of the test rig are shown in Figure 5. The test rig consists of 8 parts: servo motor, coupling, dynamometer, misalignment adjustment device, speed test module, vibration test module, data acquisition module, and host computer.

The servo motor is an SMA13-46P1B servo motor produced by Monde Electric Co., Ltd. The rated speed of the motor is 2000 rpm, and the motor's rated torque is 11 Nm. The speed accuracy of the motor is 0.1 rpm, and the motor's speed range is 0–10400 rpm. The coupling is an Oldham coupling, with an allowable radial misalignment of 2 mm and an allowable angular misalignment of 3°. The eddy current dynamometer is used to provide load, and the rated torque of the dynamometer is 10 Nm. The misalignment adjustment device is shown in Figure 3, which consists of 4 sets of cages and adjustment knobs. When adjusting the parallel misalignment, the adjustment knobs on the same side rotate simultaneously, resulting in parallel misalignment. When adjusting the angular misalignment, adjust a single knob on the same side to produce angular misalignment. The vibration test module includes two accelerometers perpendicular to each other. The vibration signal is sampled at equiangular spacing. The accelerometer is a precision quartz shear integrated circuit piezoelectricity (ICP) type with a sensitivity of 1000 mv/g and a frequency range of 0.05 o 25 kHz. Each measurement's output of the two acceleration sensors will be recorded simultaneously. The angle encoder of the motor realizes the speed test module, and the speed signal also adopts the equiangular spacing sampling method. The data acquisition module adopts the PXIe7961 acquisition system produced by NI Company, which collects the angle encoder and accelerometer signals. The speed signal acquisition accuracy of the PXIe acquisition system is 0.1 rpm.

Furthermore, the PXIe acquisition system can realize sampling with variable sampling frequencies from 0 Hz–160 MHz. For different speeds, the speed signal and the vibration signal are collected at different sampling rates to ensure that the signal is sampled once per degree. So, the sampling rate of the acquisition system is always 360 times the speed.

### 5.2. Experimental Process

The whole experiment can be divided into two parts: no-load pattern and load pattern. The practical steps of each part can be divided into the following three steps: first, the initial position adjustment of the test rig, then the driving and loading parameter settings, and finally, set the parameters for the misalignment experiment.

Before adjusting the initial position of the test rig, connect the components of the test rig described in Section 5.1 in order. Adjust the initial position of the test rig based on the double dial indicator method. Two TESA probes are used to adjust the misalignment error between the motor output shaft and the dynamometer input shaft to within 10 *μ*m, which is used as a parameter setting under the alignment condition.

After the adjustment of the test rig, the driving and loading parameters need to be set. Two experiments of no-load pattern and load pattern are set, respectively, to verify the accuracy and robustness of the diagnosis method in this paper. The no-load design adjusts the dynamometer to no-load mode (0 Nm). Adjust the drive motor to speed mode, the motor speed gradually increases from 0 rpm to 1200 rpm, and the speed increment is 300 rpm. The parameter setting of the drive motor in the load pattern is the same as that in the no-load pattern. However, the dynamometer is adjusted to torque loading mode. The load is 10 Nm.

After completing the above adjustment and parameter setting, the next step is to set the misalignment parameters for the experiment. Parameter adjustment of parallel misalignment test: adjust the parallel misalignment parameters based on the double dial indicator method. Using the misalignment adjustment device on the test rig, rotate the adjustment knob on the same side simultaneously. Furthermore, set the adjustment amount of the misalignment error between the motor output shaft and the dynamometer input shaft to 0.25 mm, 0.5 mm, 0.75 mm, 1 mm, 1.25 mm, 1.5 mm, 1.75 mm, and 2 mm, respectively. Parameter adjustment of angular misalignment test: adjust the parallel misalignment parameters based on the double dial indicator method. Using the misalignment adjustment device on the experimental bench, rotate the single adjustment knob on the same side. Moreover, set the adjustment amount of the misalignment error between the motor output shaft and the dynamometer input shaft to 0.41 mm, 0.82 mm, 1.23 mm, 1.64 mm, 2.05 mm, 2.46 mm, 2.87 mm, and 3.28 mm, respectively, which correspond to the angular misalignment 0.25°°, 0.5°°, 0.75°°, 1°°, 1.25°°, 1.5°°, 1.75°°, and 2°°, respectively.

The equiangular spacing sampling method is used in this paper. Then, the four steps described in Section 4.3 were used to obtain the final relationship model between the misalignment and the amplitude of the *f*_*r*_ component in the speed signal. After getting the relationship, the amplitude of the *f*_*r*_ component and the relationship were used to diagnose the values of misaligned malfunction quantitatively.

### 5.3. Experimental Results and Analysis

The experimental results can be divided into three parts: firstly, the diagnosis results of order spectrum analysis and the SVD-HT method are compared to verify the accuracy of the SVD-HT signal processing algorithm. Secondly, the misaligned malfunction under the no-load pattern is quantitatively diagnosed using the proposed CSF-PPSO-ESN method. Finally, the misaligned malfunction under load patterns is analyzed using the proposed CSF-PPSO-ESN method.

#### 5.3.1. Diagnosis Results of Different Signal Processing Algorithms

**Figure 6:**
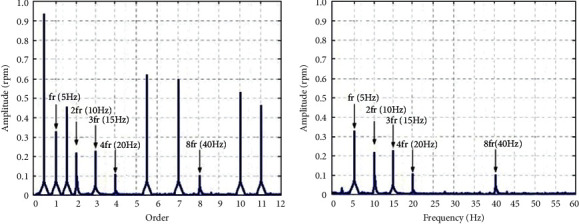
Comparison of speed signals under 1 mm parallel misalignment at 300 rpm: (a) order spectrum analysis; (b) SVD-HT method.

In order to verify the accuracy of the SVD-HT method, order spectrum analysis and the SVD-HT method are used to process the speed signal of 1 mm parallel misalignment at 300 rpm under no-load conditions.  Figure 6 shows the comparison results of speed signals of 1 mm parallel misalignment at 300 rpm under no-load conditions.

Figure 6(a) shows the diagnosis results of order spectrum analysis. It can be seen that the order spectrum of the speed signal contains more periodic noise signals. The actual malfunction signal is not dominant in the spectrum, which leads to the error in diagnosis results.  Figure 6(b) shows the diagnosis results of the SVD-HT method. After SVD-HT processing, the noise signal in the signal spectrum is suppressed. The malfunction signal and the malfunction frequency multiplier signal are consistent with the experimental setting parameters. The comparison result verifies the accuracy of the SVD-HT signal processing algorithm.

#### 5.3.2. Diagnosis Results under No-Load Pattern

The misaligned malfunction under the no-load pattern is quantitatively diagnosed in the experiments using the proposed CSF-PPSO-ESN method. The speed of the drive motor increases from 0 rpm to 1200 rpm, and the speed increment is 300 rpm. The parallel misalignment parameters are 0 mm, 0.25 mm, 0.5 mm, 0.75 mm, 1 mm, 1.25 mm, 1.5 mm, 1.75 mm, and 2 mm, respectively, and the angular misalignment parameters are 0°, 0.25°°, 0.5°°, 0.75°°, 1°°, 1.25°°, 1.5°°, 1.75°°, and 2°°, respectively.

The ESN neural network simulation results: Sixty experiments were carried out under the experimental conditions described in this paper. The first 50 groups of experimental data were used to train the neural network, and the last ten groups of experimental data were used to verify the effectiveness of the proposed CSF-PPSO-ESN method. The parameter setting of the reserve pool of the CSF-PPSO-ESN neural network is shown in Table 1.

The root mean square error (RMSE) and symmetric mean absolute percentage error (SMAPE) are the evaluation indexes of prediction performance. The average deviation of the predicted value of the proposed method from the real misaligned malfunction value was represented by the RMSE. The calculation formula is as follows:

(46) $$\begin{array}{c} \text{RMSE}=\sqrt{\overset{\text{ N}}{\underset{\text{k}=1}{\sum }}\frac{{\left(m_{k}^{\prime }-m_{k}\right)}^{2}}{\left(N-1\right)}}, \end{array}$$

where *m*_*k*_′ is the prediction value of the model,  *m*_*k*_ is the real misaligned malfunction value, and *N* is the number of samples.

The SMAPE is used to evaluate the accuracy of the proposed method and mainly evaluates the trend of the misaligned malfunction value. The calculation formula is as follows:

(47) $$\begin{array}{c} \text{SMAPE}=\frac{1}{\text{N}}\overset{\text{N}}{\underset{\text{k}=1}{\sum }}\left|\frac{m_{k}-m_{k}^{\prime }}{m_{k}+m_{k}^{\prime }}\right|. \end{array}$$

**Figure 7:**
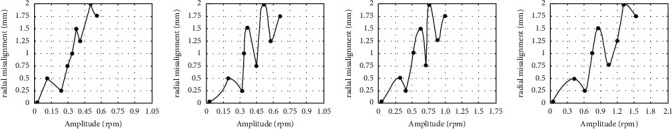
The prediction *f*_*r*_ component-parallel misalignment curve under no-load condition: (a) 300 rpm; (b) 600 rpm; (c) 900 rpm; (d) 1200 rpm.

**Figure 8:**
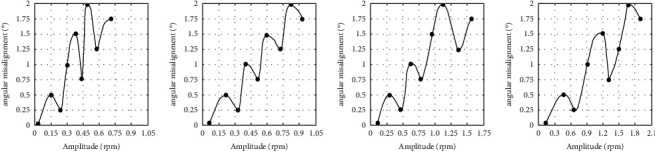
The prediction *f*_*r*_ component-angular misalignment curve under no-load condition: (a) 300 rpm; (b) 600 rpm; (c) 900 rpm; (d) 1200 rpm.

The prediction *f*_*r*_ component-parallel misalignment and *f*_*r*_ component-angular misalignment curve at 300 rpm, 600 rpm, 900 rpm, and 1200 rpm using the CSF-PPSO-ESN neural network are shown in Figures 7 and 8, respectively. From these curves, it can be seen that the prediction of the *f*_*r*_ component-misalignment curve is smooth and has prominent nonlinear characteristics.

The prediction error of the misalignment on the test set using the CSF-PPSO-ESN method is shown in Table 2. It can be seen that the deviation between the predicted value and the actual value is slight, and the RMSE reaches the order of 10^−6^. The SMAPE of the CSF-PPSO-ESN method proposed in this paper reaches the order of 10^−7^, which shows that this method can estimate the variation trend of misalignment with high accuracy. The diagnosis accuracy of parallel misalignment and angular misalignment using the proposed CSF-PPSO-ESN neural network was verified through the prediction of ten groups of data [36]. Three groups of prediction results of the parallel misalignment and angular misalignment at the speed of 1200 rpm are shown in Tables 3 and 4, respectively. It can be seen from those two tables that the diagnosing error of the shaft misalignment malfunction can reach less than ±10 *μ*m. For comparison, the order spectrum analysis and RBF neural network based on speed analysis were used to diagnose the values of misaligned malfunction under no-load conditions. Three groups of prediction results of the parallel misalignment and angular misalignment using the order spectrum analysis and RBF neural network at the speed of 1200 rpm are shown in Tables 3 and 4, respectively. It can be seen from those two tables that the diagnosing error of the shaft misalignment malfunction using the order spectrum analysis and RBF neural network can reach nearly ±50 *μ*m.

#### 5.3.3. Diagnosis Results under Load Pattern

In the experiments, the final relationship model between the misalignment and the *f*_*r*_ component's amplitude was obtained under a load pattern. The speed of the drive motor increases from 0 rpm to 1200 rpm, and the speed increment is 300 rpm. The parallel misalignment parameters are 0 mm, 0.25 mm, 0.5 mm, 0.75 mm, 1 mm, 1.25 mm, 1.5 mm, 1.75 mm, and 2 mm, respectively, and the angular misalignment parameters are 0°, 0.25°°, 0.5°°, 0.75°°, 1°°, 1.25°°, 1.5°°, 1.75°°, and 2°°, respectively.

Sixty experiments were carried out under the experimental conditions described in this paper. The first 50 groups of experimental data were used to train the neural network, and the last ten groups of experimental data were used to verify the effectiveness of the proposed CSF-PPSO-ESN method. The ESN neural network simulation results were compared with the real misaligned malfunction value to ascertain the effectiveness of the proposed method. The parameter setting of the reserve pool of the CSF-PPSO-ESN neural network is shown in Table 5.

**Figure 9:**
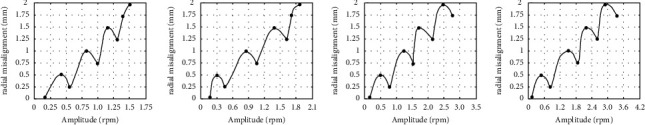
The prediction *f*_*r*_ component-parallel misalignment curve under load condition: (a) 300 rpm; (b) 600 rpm; (c) 900 rpm; (d) 1200 rpm.

**Figure 10:**
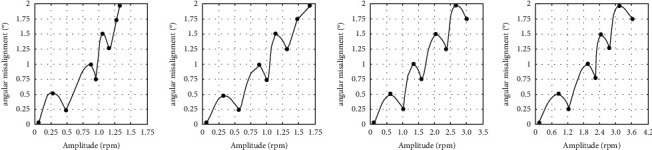
The prediction *f*_*r*_ component-angular misalignment curve under load condition: (a) 300 rpm; (b) 600 rpm; (c) 900 rpm; (d) 1200 rpm.

The prediction *f*_*r*_ component-parallel misalignment and *f*_*r*_ component-angular misalignment curve at 300 rpm, 600 rpm, 900 rpm, and 1200 rpm using the CSF-PPSO-ESN neural network are shown in Figures 9 and 10, respectively. From these curves, it can be seen that the prediction of the *f*_*r*_ component-misalignment curve is smooth and has prominent nonlinear characteristics.

The prediction error of the misalignment on the test set using the CSF-PPSO-ESN method is shown in Table 6. It can be seen that the deviation between the predicted value and the actual value is slight, and the RMSE reaches the order of 10^−6^. The SMAPE of the CSF-PPSO-ESN method proposed in this paper reaches the order of 10^−7^, which shows that this method can estimate the variation trend of misalignment with high accuracy. The diagnosis accuracy of parallel misalignment and angular misalignment using the proposed CSF-PPSO-ESN neural network was verified through the prediction of ten groups of data [36]. Three groups of prediction results of the parallel misalignment and angular misalignment at the speed of 1200 rpm are shown in Table 7, respectively. It can be seen from those two tables that the diagnosing error of the shaft misalignment malfunction in Table 8can reach less than ±10 *μ*m. For comparison, the order spectrum analysis and RBF neural network based on speed analysis were used to diagnose the values of misaligned malfunction under load conditions. Three groups of prediction results of the parallel misalignment and angular misalignment using the order spectrum analysis and RBF neural network at the speed of 1200 rpm are shown in Tables 7 and 8, respectively. It can be seen from those two tables that the diagnosing error of the shaft misalignment malfunction using the order spectrum analysis and RBF neural network can reach nearly ±50 *μ*m.

## 6. Conclusion

A novel method is proposed in this paper for quantitatively diagnosing the shaft misaligned malfunction. This method combines the motor speed signal with SVD-HT method and CSF-PPSO-ESN method. The acquisition of speed signal is realized by the motor's angle encoder, which reduces the cost of condition monitoring. The equipment condition is overall viewed, and the malfunction diagnosis mechanism based on the speed signal is obtained by constructing the shaft misalignment malfunction model.

Combining the advantages of the CSF-PPSO method and ESN neural network, the CSF-PPSO-ESN method can approach the nonlinear characteristics of the *f*_*r*_ component-misalignment curve with high accuracy, and the algorithm is simple and efficient. Therefore, compared with a single method, the proposed method has a broader scope of application. The prediction results show that the CSF-PPSO-ESN prediction model accurately describes the dynamic characteristics of the misalignment. Furthermore, the diagnosing error of the shaft misalignment malfunction can reach less than ±10 *μ*m. Through comparison, it can be seen that the diagnosing accuracy of the CSF-PPSO-ESN method is better than the accuracy of the order spectrum analysis and RBF neural network. In summary, the diagnosis method proposed in this paper can effectively realize the quantitative diagnosis of the type and value of misalignment malfunction parameters for any rotation machine.

The method proposed in this paper could be adapted and used to improve control and digital twin systems in the future. Its effects can be found in the following three parts. First, just as Toro presented the network-based identification technique [37], the CSF-PPSO-ESN method can obtain third-order models based on the recorded input/output data. Then, just as the framework proposed by Villalonga et al. [38], the CSF-PPSO-ESN method can be used to make decisions autonomously for the digital twin systems. Last but not least, the digital twins' online diagnosis system can be realized using the proposed method, just as the fast orthogonal search submitted by Peng and Chen [39].

## Figures and Tables

**Table 1:** Parameters of the reserve pool.

Size	SD (%)	Spectral radius	Input scale	Regular termcoefficient
500	5	0.9	0.1	10^–10^

**Table 2:** Prediction error.

Error type	RMSE	SMAPE
Error value	3.0264 × 10^−6^	3.3689 × 10^−7^

**Table 3:** Three groups of verification––repeating experimental data of parallel misalignment.

Standard parallel misalignment (mm)	The misaligned malfunction diagnosis results using the CSF-PPSO-ESN method (mm)			The misaligned malfunction diagnosis results using the order spectrum analysis and RBF neural network (mm)		
	Group one	Group two	Group three	Group one	Group two	Group three
0.001	0.010	0.001	0.005	0.048	0.005	0.014
0.252	0.250	0.251	0.254	0.217	0.268	0.247
0.501	0.510	0.501	0.496	0.481	0.493	0.537
0.749	0.754	0.759	0.758	0.752	0.784	0.747
1.000	1.004	1.005	0.993	0.954	0.998	0.997
1.251	1.251	1.255	1.247	1.286	1.207	1.248
1.499	1.496	1.502	1.504	1.539	1.512	1.492
1.751	1.755	1.748	1.746	1.742	1.776	1.714
2.000	1.993	2.007	1.992	2.036	1.986	1.957

**Table 4:** Three groups of verification—repeating experimental data of angular misalignment.

Standard angular misalignment (°)	The misaligned malfunction diagnosis results using the CSF-PPSO-ESN method (°)			The misaligned malfunction diagnosis results using the order spectrum analysis and RBF neural network (°)		
	Group one	Group two	Group three	Group one	Group two	Group three
0.001	0.004	0.008	0.002	0.043	0.015	0.007
0.252	0.254	0.249	0.253	0.272	0.241	0.218
0.501	0.507	0.498	0.497	0.452	0.476	0.517
0.749	0.749	0.748	0.752	0.747	0.789	0.715
1.000	0.997	1.002	0.996	0.987	1.038	0.963
1.251	1.249	1.252	1.255	1.221	1.253	1.272
1.499	1.502	1.498	1.503	1.537	1.506	1.468
1.751	1.748	1.751	1.754	1.784	1.716	1.749
2.000	2.003	1.997	2.002	2.028	2.008	1.974

**Table 5:** Parameters of the reserve pool.

Size	SD (%)	Spectral radius	Input scale	Regular term coefficient
300	5	0.8	0.2	10^–10^

**Table 6:** Prediction error.

Error type	RMSE	SMAPE
Error value	2.0264 × 10^−6^	1.3689 × 10^−7^

**Table 7:** Three groups of verification—repeating experimental data of parallel misalignment.

Standard parallel misalignment (mm)	The misaligned malfunction diagnosis results using the CSF-PPSO-ESN method (mm)			The misaligned malfunction diagnosis results using the order spectrum analysis and RBF neural network (mm)		
	Group one	Group two	Group three	Group one	Group two	Group three
0.001	0.006	0.009	0.001	0.034	0.055	0.027
0.252	0.249	0.248	0.251	0.275	0.224	0.254
0.501	0.498	0.506	0.502	0.458	0.523	0.499
0.749	0.748	0.749	0.753	0.794	0.749	0.725
1.000	0.996	0.995	1.003	0.954	0.998	1.043
1.251	1.249	1.252	1.246	1.298	1.250	1.208
1.499	1.506	1.498	1.502	1.459	1.495	1.542
1.751	1.749	1.752	1.754	1.795	1.723	1.748
2.000	2.003	1.997	1.998	1.996	1.952	2.043

**Table 8:** Three groups of verification—repeating experimental data of angular misalignment.

Standard angular misalignment (°)	The misaligned malfunction diagnosis results using the CSF-PPSO-ESN method (°)			The misaligned malfunction diagnosis results using the order spectrum analysis and RBF neural network (°)		
	Group one	Group two	Group three	Group one	Group two	Group three
0.001	0.006	0.003	0.007	0.014	0.035	0.047
0.252	0.249	0.251	0.254	0.215	0.274	0.234
0.501	0.498	0.503	0.506	0.488	0.453	0.539
0.749	0.752	0.751	0.748	0.714	0.799	0.755
1.000	1.003	0.996	1.004	1.048	0.958	1.003
1.251	1.252	1.248	1.253	1.258	1.290	1.212
1.499	1.497	1.504	1.496	1.508	1.456	1.545
1.751	1.752	1.747	1.751	1.714	1.781	1.753
2.000	1.996	2.004	1.998	1.962	2.032	2.013

## Data Availability

The data used to support the findings of this study are available from the corresponding author upon request.

## References

[1] Feng Z., Gao A., Li K., Ma H. (2021). Planetary gearbox fault diagnosis via rotary encoder signal analysis. *Mechanical Systems and Signal Processing*.

[2] Verucchi C., Bossio J., Bossio G., Acosta G. (2016). Misalignment detection in induction motors with flexible coupling by means of estimated torque analysis and MCSA. *Mechanical Systems and Signal Processing*.

[3] Lei Y., He Z., Zi Y., Chen X. (2008). New clustering algorithm-based fault diagnosis using compensation distance evaluation technique. *Mechanical Systems and Signal Processing*.

[4] Janssens O., Slavkovikj V., Vervisch B. (2016). Convolutional neural network based malfunction detection for rotating machinery. *Journal of Sound and Vibration*.

[5] Du W., Tao J., Li Y., Liu C. (2014). Wavelet leaders multifractal features based fault diagnosis of rotating mechanism. *Mechanical Systems and Signal Processing*.

[6] Nembhard A. D., Sinha J. K., Yunusa-Kaltungo A. (2015). Experimental observations in the shaft orbits of relatively flexible machines with different rotor related faults. *Measurement*.

[7] Jalan A. K., Mohanty A. R. (2009). Model based fault diagnosis of a rotor–bearing system for misalignment and unbalance under steady-state condition. *Journal of Sound and Vibration*.

[8] Yang W., Tavner P. J. (2009). Empirical mode decomposition, an adaptive approach for interpreting shaft vibratory signals of large rotating machinery. *Journal of Sound and Vibration*.

[9] Wang G., Zhao B., Xiang L., Li W., Zhu C. (2021). Information interval spectrum: a novel methodology for rolling-element bearing diagnosis. *Measurement*.

[10] Wang Z., Yao L., Chen G., Ding J. (2021). Modified multiscale weighted permutation entropy and optimized support vector machine method for rolling bearing fault diagnosis with complex signals. *ISA Transactions*.

[11] Tavasolipour E., Poshtan J., Shamaghdari S. (2021). A new approach for robust fault estimation in nonlinear systems with state-coupled disturbances using dissipativity theory. *ISA Transactions*.

[12] Su H., Xiang L., Hu A., Gao B., Yang X. (2021). A novel hybrid method based on KELM with SAPSO for fault diagnosis of rolling bearing under variable operating conditions. *Measurement*.

[13] Xu Z., Li C., Yang Y. (2021). Fault diagnosis of rolling bearings using an improved multi-scale convolutional neural network with feature attention mechanism. *ISA Transactions*.

[14] Kim M., Ko J. U., Lee J., Youn B. D., Jung J. H., Sun K. H. (2022). A Domain Adaptation with Semantic Clustering (DASC) method for fault diagnosis of rotating machinery. *ISA Transactions*.

[15] Meng J., Wang H., Zhao L., Yan R. (2021). Compound fault diagnosis of rolling bearing using PWK-sparse denoising and periodicity filtering. *Measurement*.

[16] Gonçalves J. P., Fruett F., Dalfré Filho J. G., Giesbrecht M. (2021). Malfunctions detection and classification in a centrifugal pump from vibration data using Markov parameters. *Mechanical Systems and Signal Processing*.

[17] Chen X., Qi X., Wang Z., Cui C., Wu B., Yang Y. (2021). Fault diagnosis of rolling bearing using marine predators algorithm-based support vector machine and topology learning and out-of-sample embedding. *Measurement*.

[18] Zhang K., Tang B., Deng L., Liu X (2021). A hybrid attention improved ResNet based fault diagnosis method of wind turbines gearbox. *Measurement*.

[19] Khan M. A., Shahid M. A., Ahmed S. A. (2019). Gear misalignment diagnosis using statistical features of vibration and airborne sound spectrums. *Measurement*.

[20] Wang L., Yun F., Yao S., Liu J. (2016). Measurement method and pipe wall misalignment adjustment algorithm of the pipe butting machine. *Measurement*.

[21] Patel T. H., Darpe A. K. (2009). Experimental investigations on vibration response of misaligned rotors. *Mechanical Systems and Signal Processing*.

[22] Jung J. H., Jeon B. C., Youn B. D., Kim M., Kim D., Kim Y. (2017). Omnidirectional regeneration (ODR) of proximity sensor signals for robust diagnosis of journal bearing systems. *Mechanical Systems and Signal Processing*.

[23] Yu Z., Zhang Y. (2021). Diagnosis of the coupling misalignment of the vertical comprehensive performance test instrument of high precision reducer for industrial robot. *Measurement*.

[24] Yang M., Chai N., Liu Z., Ren B., Xu D. (2019). Motor speed signature analysis for local bearing malfunction detection with noise cancellation based on improved drive algorithm. *IEEE Transactions on Industrial Electronics*.

[25] Chandra M., Sekhar A. S. (2015). Detection and monitoring of coupling misalignment in rotors using torque measurements. *Measurement*.

[26] Prakht V. A., Dmitrievskii V. A., Ka Zakbaev V. M., Oshurbekov S. K. (2020). Comparative analysis of two high-speed single-phase electrical machines with permanent magnet on the stator. *Electrical Engineering & Electromechanics*.

[27] Cruz Y. J., Rivas M., Quiza R., Villalonga A., Haber R. E., Beruvides G. (2021). Ensemble of convolutional neural networks based on an evolutionary algorithm applied to an industrial welding process. *Computers in Industry*.

[28] Tirkolaee E. B., Goli A., Faridnia A., Soltani M., Weber G. W. (2020). Multi-objective optimization for the reliable pollution-routing problem with cross-dock selection using Pareto-based algorithms. *Journal of Cleaner Production*.

[29] He C., Li L., Tian Y. (2019). Accelerating large-scale multiobjective optimization via problem reformulation. *IEEE Transactions on Evolutionary Computation*.

[30] Avina-Corral V., Rangel-Magdaleno J., Morales-Perez C., Hernandez-Perez J. (2021). Bearing malfunction detection in ASD-powered induction machine by using MCSA and goodness-of-fit tests. *IEEE Transactions on Industrial Informatics*.

[31] Mingjun G., Weiguang L., Qijiang Y., Zhao X., Tang Y. (2020). Amplitude filtering characteristics of singular value decomposition and its application to malfunction diagnosis of rotating machinery. *Measurement*.

[32] Hanus R. (2019). Time delay estimation of random signals using cross-correlation with Hilbert Transform. *Measurement*.

[33] Yang J., Zhou J., Liu L., Li Y. (2009). A novel strategy of Pareto-optimal solution searching in multi-objective particle swarm optimization (MOPSO). *Computers & Mathematics with Applications*.

[34] Jane C. J. (2014). An application of Pareto particle swarm optimization using with geographic information system technology. *International Journal of Kansei Information*.

[35] Lemos T., Campos L. F., Melo A. (2021). Echo state network based soft sensor for monitoring and fault detection of industrial processes. *Computers & Chemical Engineering*.

[36] Yu Z., Qiu Z., Li H., Xue J., Hu W., Wang C. (2022). Design and calibration of torque measurement system of comprehensive performance test instrument of industrial robot reducer. *Computational Intelligence and Neuroscience*.

[37] Toro R. M. d., Schmittdiel M. C., Haber-Guerra R. E., Haber R. H. System identification of the high performance drilling process for network-based control.

[38] Villalonga A., Negri E., Biscardo G. (2021). A decision-making framework for dynamic scheduling of cyber-physical production systems based on digital twins. *Annual Reviews in Control*.

[39] Peng C. C., Chen Y. H. (2022). Digital twins-based online monitoring of TFE-731 turbofan engine using Fast orthogonal search. *IEEE Systems Journal*.

